# Recent Advances in the Diagnosis and Management of Multiple Primary Lung Cancer

**DOI:** 10.3390/cancers14010242

**Published:** 2022-01-04

**Authors:** Chi-Lu Chiang, Ping-Chung Tsai, Yi-Chen Yeh, Yuan-Hung Wu, Han-Shui Hsu, Yuh-Min Chen

**Affiliations:** 1 Department of Chest Medicine, Taipei Veterans General Hospital, Taipei 112, Taiwan; clchiang@vghtpe.gov.tw; 2 School of Medicine, National Yang Ming Chiao Tung University, Taipei 112, Taiwan; lordaaa@gmail.com (Y.-C.Y.); yhwu13@vghtpe.gov.tw (Y.-H.W.); hsuhs@vghtpe.gov.tw (H.-S.H.); 3 Institute of Clinical Medicine, National Yang Ming Chiao Tung University, Taipei 112, Taiwan; 4 Department of Surgery, Division of Thoracic Surgery, Taipei Veterans General Hospital, Taipei 112, Taiwan; pctsai9@vghtpe.gov.tw; 5 Department of Pathology and Laboratory Medicine, Taipei Veterans General Hospital, Taipei 112, Taiwan; 6 Department of Oncology, Division of Radiation Oncology, Taipei Veterans General Hospital, Taipei 112, Taiwan; 7 Department of Biomedical Imaging and Radiological Sciences, National Yang Ming Chiao Tung University, Taipei 112, Taiwan

**Keywords:** multiple primary lung cancer, next-generation sequencing, surgery, stereotactic ablative radiotherapy, immunotherapy, targeted therapy

## Abstract

**Simple Summary:**

With the wide application of computed tomography and lung cancer screening, the incidence of multiple primary lung cancer, that is, the occurrence of two or more primary malignant lung tumors in a patient, has been increasingly reported. The optimal strategy for the diagnosis and treatment of multiple primary lung cancers is controversial. Surgery remains the main treatment modality, whereas other treatment methods, including radiotherapy and local ablation, are also feasible, particularly for inoperable patients. Next-generation sequencing and novel therapies, such as targeted agents and immune-checkpoint inhibitors, have provided new insights into this topic.

**Abstract:**

With the wide application of computed tomography in lung cancer screening, the incidence of multiple primary lung cancer (MPLC) has been increasingly reported. Despite the established criteria, the differentiation between MPLC and intrapulmonary metastasis remains challenging. Although histologic features are helpful in some circumstances, a molecular analysis is often needed. The application of next-generation sequencing could aid in distinguishing MPLCs from intrapulmonary metastasis, decreasing ambiguity. For MPLC management, surgery with lobectomy is the main operation method. Limited resection does not appear to negatively affect survival, and it is a reasonable alternative. Stereotactic ablative radiotherapy (SABR) has become a standard of care for patients refusing surgery or for those with medically inoperable early-stage lung cancer. However, the efficacy of SABR in MPLC management could only be found in retrospective series. Other local ablation techniques are an emerging alternative for the control of residual lesions. Furthermore, systemic therapies, such as targeted therapy for oncogene-addicted patients, and immunotherapy have shown promising results in MPLC management after resection. In this paper, the recent advances in the diagnosis and management of MPLC are reviewed.

## 1. Introduction

Lung cancer is the leading cause of cancer-related death worldwide [1]. With the advances in modern treatment, the 2-year survival rate has increased from 34% to 42% in patients with non-small cell lung cancer (NSCLC) [1]. Moreover, the diagnosis of multiple primary lung cancer (MPLC) is increasing due to the use of computed tomography scanning [2]. MPLC is classified into two types, namely synchronous MPLC (sMPLC) and metachronous MPLC (mMPLC), according to the time of lesion occurrence. Despite the established criteria, distinguishing between MPLC and intrapulmonary metastasis (IM) is still challenging sometimes [3,4,5,6]. Positron emission tomography–computed tomography (PET–CT) could aid the differential diagnosis in the absence of histology [7,8,9].

Most patients with MPLC are diagnosed with multiple ground glass opacities (GGOs) simultaneously, and therefore, surgery is the mainstay of management in the early stages of the disease [10,11]. The proportion of patients with sMPLC and 5-year survival rate is increasing, whereas the postoperative mortality is decreasing [12]. A previous meta-analysis revealed that age, sex, pulmonary function, smoking, tumor size, surgical methods, and lymph node status are prognostic factors for sMPLC [12].

With the increase in life expectancy, some patients with lung cancer develop a second primary lung malignancy, namely mMPLC, with a cumulative incidence of approximately 20% in both never-smokers and ever-smokers following surgical resection [13]. Repeat surgery may not be feasible due to compromised pulmonary function. Therefore, effective intervention other than surgery is needed for managing this patient group.

The diagnosis and management of MPLC have greatly improved recently due to the advancement of novel technologies and therapies, particularly next-generation sequencing (NGS). In this paper, we review relevant English language journal articles indexed in PubMed as of October 2021 (using the search terms “multiple primary lung cancer” OR “multiple ground-glass opacities”) and discuss the recent advances in this field.

## 2. Pathological and Molecular Perspective

### 2.1. Histologic Interpretation of MPLC and IM

Histologic distinction between MPLC and IM is challenging. Histologic features can be helpful in several circumstances. First, tumor pairs of different histologic types, for example, squamous cell carcinoma and adenocarcinoma, are considered different primary tumors. Second, multiple foci of adenocarcinoma in situ, minimally invasive adenocarcinoma, and lepidic adenocarcinoma are also considered different primary tumors [6]. Third, tumors with a precursor lesion or carcinoma in situ component, such as squamous cell carcinomas with adjacent squamous cell carcinoma in situ or adenocarcinomas with lepidic component, are generally considered different primary lung cancers [14]. However, such criteria should be used with caution. For example, in some adenocarcinomas, a lepidic pattern may represent outgrowth/surface colonization of an invasive tumor, rather than a precursor lesion [15,16]. Therefore, the presence of the lepidic component itself, particularly when present in limited areas, does not preclude the possibility of IM. Fourth, for invasive mucinous adenocarcinomas, recent molecular studies have shown that nearly all separate pulmonary invasive mucinous adenocarcinomas represent IM from a single tumor, although rare exceptions do occur [17,18]. Finally, for invasive non-mucinous adenocarcinomas, the current American Joint Committee on Cancer staging system recommends using a comprehensive histologic assessment to differentiate primary lung cancers from IM [6,19]. A comprehensive histologic assessment refers to a histologic comparison between different lung adenocarcinomas through a detailed evaluation of predominant and minor histologic patterns, as well as cytologic and stromal features. A recent study conducted by the International Association for the Study of Lung Cancer Pathology Committee investigated the ability and reproducibility of pathologists to apply this approach [14]. The result revealed a good interobserver agreement among pathologists in the assessment of separate primary lung cancers from IM, with a Cohen kappa statistic of 0.596 and overall agreement of 81%. However, such agreement is still far from perfect. Additionally, up to 25.5% of cases might be regarded as “uncertain” and not be classified into MPLCs or IM [14]. These results indicate a need to improve the performance of the current classification approach based on histology.

### 2.2. Molecular Analysis of MPLC and IM

Over the past decades, many studies have investigated the use of molecular analysis methods in the differentiation between MPLCs and IM, including DNA microsatellite analysis, TP53 mutation analysis, array comparative genomic hybridization, oncogenic driver hotspot mutation testing, genomic breakpoint analysis, and most recently NGS [15,20,21,22,23,24,25,26,27,28,29,30]. Among these approaches, targeted NGS panels have received the highest research attention because genomic profiling with NGS panels has been widely used in clinical practice in lung cancer. NGS enables a simultaneous investigation of mutations in numerous genes, including oncogenic driver mutations and other co-occurring mutations. In treatment-naïve lung cancer, oncogenic driver mutations, such as epidermal growth factor receptor (EGFR), Kirsten rat sarcoma virus (KRAS), BRAF, anaplastic lymphoma kinase (ALK), and ROS1, typically occur as trunk mutations with little intratumoral heterogeneity [31]. A different mutation profile in oncogenic driver mutations strongly indicates separate clonal origins. However, the presence of an identical driver mutation does not necessarily indicate tumors of clonal origin. Shared constitutional genetic background and environmental exposure may result in multiple independent primary lung cancers with identical KRAS or EGFR mutations [25,32]. Therefore, testing on a small set of oncogenic driver mutations is insufficient to distinguish between primary lung cancers and IM. Studies using large-scale NGS panels are generally more capable of distinguishing primary lung cancers from IM, with fewer ambiguities. A recent study used the 341–468 gene Memorial Sloan Kettering-Integrated Mutation Profiling of Actionable Cancer Targets (MSK-IMPACT) large-scale NGS assay to investigate molecular alterations in the pairs of multiple lung cancers [15]. Tumor pairs exhibiting entirely nonoverlapping, unique mutations were classified as MPLCs; tumors sharing multiple (≥2) mutations were regarded as IM; for tumors sharing a single hotspot mutation, the designation of different primary lung cancers and IM was made through an extended molecular review on an individual basis. The authors found that a comprehensive NGS assay could unambiguously distinguish MPLCs from IM in all tumor pairs in their study. Notably, histologic interpretation was discordant with NGS in 22% of cases [15]. These results highlight the importance of incorporating molecular information into the clinical management of MPLCs, although the optimal approach for molecular classification of MPLCs versus IM remains unclear.

## 3. Management of MPLC

### 3.1. Surgical Treatment 

#### 3.1.1. Applicability of Lobectomy

The management of MPLC should be based on the judgment of a multidisciplinary team, and thorough work-up to rule out N2~3 or M1b disease. Invasive mediastinal staging and extrathoracic imaging should be done for pretreatment evaluation. Surgical approach is the first choice recommended by the American College of Chest Physicians for those with a new primary tumor [4], but the optimal surgical strategy for MPLC remains controversial. In the most recent decade, several studies have been struggling to provide sufficient evidence for a suitable solution for MPLCs (Table 1). In a previous study, the survival of 26 patients surgically treated with sMPLC was analyzed [33]. A trend toward poor survival was presented in patients who underwent pneumonectomy, but no survival disadvantage was observed in those who underwent sublobar resection. Aggressive surgical approach might lead to poor survival or increased surgical mortality in patients with old age and underlying comorbidities [34]. Yu et al. reviewed the survival outcomes of 97 patients with synchronous lesions [35]. Patients undergoing sublobar resection or lobectomy did not exhibit a significant difference in 5-year survival (64.7% and 79.7%, *p* = 0.331) or 5-year progression-free survival (42.9% and 62.4%, *p* = 0.312). The use of limited resection did not appear to be a significant prognostic factor for survival, and no superior outcomes were observed with extended resections as in lobectomy. Surgical strategies for lung preservation should be the main approach in this cohort. Studies have reported no difference in survival irrespective of whether limited resection was performed. Ishikawa et al. analyzed 93 patients with sMPLC; multivariate analysis revealed sublobar resection to be a significant independent predictor of poor outcomes (*p* = 0.042), which led to a negative impact on curability [36]. Although several review articles have revealed that limited resection is acceptable for patients with MPLC at an early stage, the results are controversial owing to their heterogeneity [10,37,38]. Therefore, for a more accurate surgical evaluation, experienced multidisciplinary teams should make a comprehensive decision regarding the efficacy of limited resection by collecting all relevant information.

#### 3.1.2. Differences between sMPLC and mMPLC

For the management of a second tumor as synchronous or metachronous, Zuin et al. investigated 23 (19%) and 98 (81%) patients with sMPLC and mMPLC, respectively [39]. The 5-year survival was better in the lobectomy group than in the sublobar resection group (57.5% and 36%, respectively, *p* = 0.016) for the management of second primary lung cancer. Completion pneumonectomy should only be performed in carefully selected patients. In addition, the 5-year survival was significantly better in the mMPLC group compared with the sMPLC group (83% and 40%, respectively, *p* = 0.02), which was calculated from the time of initial diagnosis [39]. By contrast, an analysis of 101 patients with stage I MPLC under curative intent revealed the effectiveness of the limited resection procedure because the contralateral second nodule in these patients did not have a negative effect on the 5-year overall survival [40]. Patients with synchronous diseases did not experience a reduced survival rate compared with those with metachronous diseases, which is in agreement with findings of previous systematic reviews and meta-analyses [10,42]. Multiple pulmonary resections are considered effective among patients with synchronous and metachronous lung cancer [43]. 

#### 3.1.3. Residual Lesions and Surveillance after Surgery

Shimada et al. accessed the survival outcomes of patients with MPLCs after surgery and revealed a residual lesion growth in 8% and new GGO development in 23% of the patients. However, neither the growth of residual GGOs nor the development of new GGOs affected post-operative survival [11]. The same research group then compared 190 patients with multifocal GGOs with those with solitary lung adenocarcinoma and revealed that the recurrence-free survival outcome was similar in the two groups. Among 116 patients with residual lesions, 38 patients exhibited progressed lesions during the follow-up period [44]. Therefore, after operation, surveillance of the unresected lesions is required. To date, the management of residual lesions that are not resected with the main tumor in the initial surgery, remains controversial. 

### 3.2. Radiation Therapy

Stereotactic ablative radiotherapy (SABR), or stereotactic body radiotherapy (SBRT) involves the integration of image-guiding and respiratory-control modalities to deliver conformal high-dose radiation to the tumor, while limiting the dose to the surrounding organs over a period of 1–2 weeks. A randomized controlled trial (RCT) conducted by the Tasman Radiation Oncology Group with variable radiotherapy doses demonstrated that SABR yields better overall survival and disease control with fewer complications compared with conventional radiotherapy [45]; however, another RCT also demonstrated fewer complications of SABR but no difference in survival or local control in early-stage lung cancer compared with the uniform dose of conventional radiotherapy [46]. SABR has become the standard-of-care for patients who refuse to undergo surgery or for those with medically inoperable early-stage lung cancer. No RCT involving operable patients has been published; however, pooled analysis of two incomplete RCTs demonstrated superior overall survival among patients who underwent SABR compared with surgery [47]. A subsequent study further demonstrated that in patients with operable stage I NSCLC, SABR had similar long-term survival outcome compared to surgery [48]. For patients with MPLCs, to preserve as much normal lung parenchyma as possible, SABR, alone or in combination with surgery, is a reasonable choice to control lung tumors. To the best of our knowledge, no RCT has compared the efficacy of SABR in the management of MPLCs with that of other local control management approaches. A literature review yielded only retrospective series on the management of sMPLC and mMPLC as shown in Table 2. Considering the lack of large-scale RCTs, the recommendation of SABR for the treatment of MPLCs by the American Society of Radiation Oncology in 2017 can be deemed acceptable [49]. Nevertheless, the board strongly recommends the evaluation of MPLCs by a multidisciplinary team and administration of PET–CT and brain magnetic resonance imaging to patients suspected of having MPLC. Moreover, SABR is strongly recommended as a curative treatment option for mMPLCs. However, for sMPLCs, SABR is only conditionally recommended as a curative treatment option. The board concluded that for sMPLCs, SABR yields equivalent rates of local control and toxicity but decreased rates of overall survival compared with those with single tumors.

### 3.3. Local Ablation Therapy

Image-guided percutaneous thermal ablation, including radiofrequency ablation, microwave ablation (MWA), and cryoablation, has been increasingly used for medically inoperable patients with early-stage lung cancer [62,63]. Recently, these techniques have also been applied in the management of MPLC with promising results [64,65]. Huang et al. reported a 100% technical success rate of treating multiple synchronous GGOs with computed-tomography–guided percutaneous MWA, with an acceptable complication rate [65]. Qu et al. further combined electromagnetic navigation bronchoscopy (ENB)-guided MWA with uniportal VATS in 11 patients with multiple GGO, and reported a 100% success rate, with no recurrence observed at follow-up [66]. Taken together, thermal ablation may also be employed as an alternative approach for treating patients with inoperable MPLC. However, studies with longer follow-up duration are warranted to evaluate the survival outcome.

### 3.4. Targeted Therapy

Targeted therapy has been successfully used in the management of lung cancer with driver oncogenes [67,68]. Reports have shown that MPLCs have a high incidence of driver mutations, such as EGFR mutations [69,70,71], around 45.8–76% in Asian patients, implying an opportunity for targeted therapies in MPLC management. Case reports have revealed that combining treatment with surgery for the main lesion and EGFR-tyrosine kinase inhibitors (TKIs) for the residual lesions may be a reasonable approach to achieve a long-term disease control [72,73]. However, some obstacles were observed. First, the tumor harboring a targetable mutation may not be representative of other lesions. The discrepancy rate of driver mutations in MPLCs is relatively high, ranging from 80% to 92.1% [70,71,74]. Moreover, the optimal treatment duration of salvage targeted therapies is unknown, and the long-term side effect profiles of these new targeted agents are also unknown. These drawbacks render the application of targeted drugs to treat MPLCs challenging.

Osimertinib, a third-generation EGFR-TKI, has demonstrated favorable efficacy against common EGFR mutation and fewer adverse effects compared with early-generation TKIs. In addition to being a new standard of care for treatment-naïve advanced EGFR mutant NSCLC, it has been proven to be effective in adjuvant settings in treating early-stage EGFR-mutant NSCLC after surgery [75]. Furthermore, the combination of osimertinib with other new targeted agents, alectinib, was reported to be effective and tolerable among patients with MPLCs with a distinct molecular profile [76]. Together, despite the heterogenicities of different tumors in MPLCs, targeted therapies, particularly EGFR-TKIs, in combination with surgery, are still a reasonable alternative strategy. Furthermore, targeted agents should be considered in the management of EGFR mutant tumors in medically inoperable patients. Further investigation is required to identify different genetic alterations in individual tumors and to tailor targeted agents for patients with MPLC.

### 3.5. Immunotherapy

Immune checkpoint inhibitors (ICIs), particularly with therapeutic antibodies targeting programmed-death ligand 1 (PD-L1)/PD-1, are currently the backbone of first-line therapy in patients with advanced NSCLC without driver oncogenes [67,68]. PD-L1 expression in tumor cells is the most important biomarker for selecting patients for treatment with ICIs [77]. However, studies regarding the PD-L1 expression level in individual tumors of patients with MPLC are scarce [78,79]. Haratake et al. retrospectively reviewed 112 MPLC lesions from 43 patients and showed that only 13.4% of lesions were PD-L1 positive. The rate of discordance in the expression of PD-L1 among MPLC patients was 27.9% [78]. Furthermore, recent studies have investigated the tumor microenvironment in MPLCs. Wu et al. analyzed the genomic and transcriptomic profiles of four tumors from two patients with MPLC and found these profiles to be considerably different. Furthermore, distinct tumor microenvironments were observed in the two tumors from the same patient [80]. Izumi et al. analyzed 73 specimens from 32 patients with MPLCs and found that woman and never/light smokers had a higher chance of PD-L1-negative tumors. The concordance rates of CD3-positive tumor-infiltrating lymphocytes and CD8/CD3 ratios was 56.2% and 53.1%, respectively [81].

The efficacy of ICIs in patients with MPLC after surgery remains controversial. Wu et al. reviewed 37 lesions with synchronous ground glass nodules (GGNS) from 18 patients in a lung adenocarcinoma cohort treated with anti-PD-1/PD-L1 therapy [82]. Despite the high response rate of primary lesions, only 8.1% of mixed GGNs responded to treatment, and 67.6% of GGNs showed no obvious change after anti-PD-1/PD-L1 treatment. The synchronous GGNs had significantly fewer CD8+ T cells and more CD68+ tumor-associated macrophages compared with primary lesions [82]. GGNs are prone to contain driver oncogenes, which is an indicator of a low response rate to ICIs [83]. By contrast, other reports have shown promising results for ICIs application in the management of patients with MPLCs [84,85]. Zhang et al. tested the efficacy of atezolizumab, an anti-PD-L1 agent, as neoadjuvant therapy in a patient with MPLC, and observed different treatment responses in each lesion [86]. Moreover, atezolizumab was recently shown to extend the disease-free survival period after surgical resection and adjuvant chemotherapy in patients with PD-L1-positive early-stage NSCLC [87]. Other trials investigating the efficacy of ICIs alone or in combination with other treatments for MPLC are still ongoing (NCT04047186, NCT04026841, NCT 04840758, and NCT05053802). In summary, the application of ICIs in the neoadjuvant or adjuvant treatment of MPLCs is challenging due to disparities in genomic alterations and the immune microenvironment among different lesions. Further studies using multiomics analysis are needed to elucidate the evolution of GGNs and the therapeutic niche of immunotherapy.

## 4. Conclusions

Rapid advances have been made in the diagnosis and management of MPLC due to the development of novel technologies. Broad-panel NGS can be used to unambiguously distinguish different primary lung carcinomas from IM; therefore, it plays a key role in MPLC diagnosis in addition to histology. Moreover, driver-oncogenes could be identified for the subsequent treatment. Surgery remains the primary treatment, and the extent of surgery should be evaluated by an experienced multidisciplinary team. SABR and local ablation therapies are reasonably safe and feasible for medially inoperable patients, but more data are needed to assess their long-term survival outcomes. Targeted therapies in combination with surgery are emergent treatment options, particularly for EGFR-mutant patients. With a better understanding of the tumor microenvironment, immunotherapies such as ICIs may also be feasible in the neoadjuvant or adjuvant setting for treating patients with MPLC. Nevertheless, further investigation is warranted to implement personalized management strategies for patients with MPLC.

## Figures and Tables

**Table 1:** Studies on the resection methods and outcomes of MPLCs.

Author, (Year)	Patient Group	Study Period	Outcome	Results	Conclusion
C.I. Kocaturk et al. (2011), [33]	Sublobar resection: *n* = 1 Lobectomy + sublobar resection: *n* = 8 Bilateral lobectomies: *n* = 3 Bilobectomy: *n* = 3 Pneumonectomy: *n* = 11 sMPLC: *n* =26	January 2001 to December 2008	Overall survival	5-year OS Pneumonectomy: 27% No-Pneumonectomy: 71.1% *p* = 0.12	Poor survival trend was observed in patients who received pneumonectomy, (*p* = 0.05, multivariate)
E.J. Jung et al. (2011), [34]	Simple lobectomy: *n* = 6 Sublobar resections: *n* = 2 Lobectomy + sublobar resection: *n* = 12 Lobectomy + PDT: *n* = 2 Bilateral lobectomies: *n* = 1 Bilobectomy: *n* = 4 Pneumonectomy: *n* = 5 sMPLC: *n* = 32	January 1995 to December 2008	Progression-free survival and overall survival	Use of limited resection 5-year OS: 79.4% 5-year PFS: 74.5% Lobectomy or extended 5-year OS: 51.2% 5-year PFS: 34.2%	The use of limited resection did not seem to negatively affect survival (multivariate analysis). Decisions regarding aggressive surgical treatments should be made carefully for older patients with underlying comorbidities owing to the poor OS and increased surgical mortality
A. Zuin et al. (2013), [39]	Second intervention Lobectomy: *n* = 61 (completion pneumonectomy: *n* =17) Atypical resection: *n* = 38 Segmentectomy: *n* = 22 sMPLC + mMPLC: *n* =121	January 1995 to December 2010	Overall survival	Lobectomy 5-year OS: 57.5% Sublobar resection 5-year OS: 36% *p* = 0.016	Lobectomy is still considered the treatment of choice in the management of second primary lung cancer, but completion pneumonectomy was a negative prognostic factor of long-term survival.
Yu et al. (2013), [35]	Sublobar resection: *n* = 14 Lobectomy + sublobar resection: *n* = 36 Lobectomy: *n* = 39 Bilateral lobectomies: *n* = 8 sMPLC: *n* = 97	January 2001 to December 2011	Progression-free survival and overall survival	5-year PFS: Sublobar resection 42.9% Lobectomy 62.4% *p* = 0.312 5-year OS: Sublobar resection: 64.7% Lobectomy 79.7% *p* = 0.331	Univariate analysis revealed no superior survival outcome among patients who underwent lobectomies compared to sublobar resections
Ishikawa et al. (2014), [36]	Sublobar resection: *n* = 27 Lobectomy + sublobar resection: *n* = 27 Lobectomy: *n* = 28 Bilateral lobectomies: *n* = 5 Bilobectomy: *n* =5 Pneumonectomy; *n* = 1 sMPLC: *n* = 93	April 1995 to December 2009	Recurrence-free survival and overall survival	Sublobar resection (OS) HR = 4.425, 95% CI 1.054–18.580, *p* = 0.042	Multivariate analysis revealed that sublobar resection was a significant independent predictor of poor outcomes
Yang et al. (2016), [40]	Sublobar resection: *n* = 13 Lobectomy + sublobar resection: *n* = 49 Bilateral lobectomies: *n* = 39 sMPLC + mMPLC: *n* =101	January 2001 to June 2014	Overall survival	5-year OS (mean, months) Lobar-lobar 70.6% Lobar-sublobar 56.7% Sublobar-sublobar 36.8%	The use of a limited resection procedure for the contralateral nodule in patients with stage I tumors did not have a negative effect on the 5-year OS.
Hattori et al. (2020), [41]	Sublobar resection: *n* = 74 Lobectomy + sublobar resection: *n* = 86 Lobectomy: *n* = 91 Bilateral lobectomies: *n* = 18 Pneumonectomy: *n* = 3 sMPLC: *n* = 272	January 2008 to December 2015	Recurrence-free survival and overall survival	OS after lobectomy HR = 1.71, 95% CI 0.494–5.920, *p* = 0.397	No clear-cut criteria exist for setting an appropriate operative mode; operative modes are essentially decided based on the radiologic findings of dominant lesions.

MPLC: multiple primary lung cancer; sMPLC: synchronous multiple primary lung cancer; mMPLC: metachronous multiple primary lung cancer; PDT: photodynamic therapy; OS: overall survival; PFS: progression-free survival; HR: hazard ratio; CI: confidence interval.

**Table 2:** Published series on the effect of SABR on sMPLC and mMPLCs.

Author (Year)	N	Treatment	Median Follow-Up (Month)	Toxicity Grade, %	Local Control	Overall Survival
sMPLCs						
Sinha et al. (2006) [50]	8	N/A	18.5	≥3, 0%	93% (1.5-years)	100% (1.5-year)
Creach et al. (2012) [51]	15	3 (OP + SABR) 12 (SABR × 2)	24	≥3, 0%	90% (at follow-up)	27.5% (2-year)
Matthiesen et al. (2012) [52]	9	8 (SABR × 2) 1 (SABR × 3)	15.5	≥2, 0%	88.9% (1.3-year)	55.5% (1.3-year)
Chang et al. (2013) [53]	39	8 (OP + SABR) 21 (SABR + SABR) 10 (cRT + SABR)	36	>3, 1% (sMPLCs + mMPLCs)	97.4% (2-year) (sMPLCs + mMPLCs)	61.5% (2-year)
Griffioen et al. (2013) [54]	62	56 (OP + SABR) 6 (OP × 2)	44	≥3, 4.8%	84% (2-year)	56% (2-year)
Rahn et al. (2013) [55]	6	N/A	20	≥2, 17% (sMPLCs + mMPLCs)	81% (2-year) (sMPLCs + mMPLCs)	62% (2-year) (sMPLCs + mMPLCs)
Kumar et al. (2014) [56]	26	SABR × 2	12	≥3, 4%	96% (at follow-up)	N/A
Shintani et al. (2015) [57]	18	3 (OP + SABR) 15 SABR × 2	34.3	≥3, 11%	78% (3-year)	69% (3-year)
Nikitas et al. (2019) [58]	14	SABR × 2	37	≥3, 14.2%	75% (3-year)	46.4% (3-year)
Miyazaki et al. (2020) [59]	26	26 (OP + SABR)	30	≥3, 3.8%	84.6% (2.5-year)	69.2% (2.5-year)
Steber et al. (2021) [60]	36	SABR × 2	51.5	≥2, 2.8%	93.4% (3-year)	63% (3-year)
mMPLCs						
Sinha et al. (2006) [50]	3	N/A	18.5	≥3, 0%	66% (1.5-year)	100% (1.5-year)
Creach et al. (2012) [51]	48	46 (OP + SABR) 2 (SABR × 2)	24	≥3, 0%	92% (at follow-up)	68.1% (2-year)
Matthiesen et al. (2012) [52]	2	2 (SABR × 3)	15.5	≥2, 0%	100% (1.3-year)	100% (1.3-year)
Chang et al. (2013) [53]	62	34 (OP + SABR) 8 (SABR × 2) 15 (cRT + SABR) 5 (OP + PORT + SABR)	36	>3, 1% (sMPLCs + mMPLCs)	97.4% (2-year) (sMPLCs + mMPLCs)	80.6% (2-year)
Griffioen et al. (2013) [54]	62	56 (OP + SABR) 6 (OP × 2)	44	≥3, 4.8%	84% (2-year)	56% (2-year)
Rahn et al. (2013) [55]	12	N/A	20	≥2, 17% (sMPLCs + mMPLCs)	81% (2-year) (sMPLCs + mMPLCs)	62% (2-year) (sMPLCs + mMPLCs)
Nishiyama et al. (2015) [61]	31	N/A	36	N/A	N/A	62% (3-year) (MPLCs + IM)
Nikitas et al. (2019) [58]	156	108 (OP + SABR) 48 (SABR × 2)	37	≥3, 5.6% (OP + SABR), 4.2% (SABR × 2)	98.2% (3-year) (OP + SABR) 96% (3-year) (SABR × 2)	79.7% (3-year)

N/A: not available, OP: operation, SABR: stereotactic ablative radiotherapy, cRT: conventional radiotherapy, PORT: post-operative radiotherapy, IM: intrapulmonary metastasis.
